# Chromosomal-scale genome assembly of the near-extinction big-head schizothorcin (*Aspiorhynchus laticeps*)

**DOI:** 10.1038/s41597-022-01671-1

**Published:** 2022-09-09

**Authors:** Jiangong Niu, Renming Zhang, Jiangwei Hu, Tao Zhang, Hong Liu, Muyit Minavar, Hui Zhang, Weiwei Xian

**Affiliations:** 1 Xinjiang Fisheries Research Institute, Urumqi, 830000 China; 2 Scientific Observing and Experimental Station of Fishery Resources and Environment in Northwest China, Ministry of Agriculture and Rural Affairs of the People’s Republic of China, Urumqi, 830000 China; 3 CAS Key Laboratory of Marine Ecology and Environmental Sciences, Institute of Oceanology, Chinese Academy of Sciences, Qingdao, 266071 China

**Keywords:** Ichthyology, Taxonomy

## Abstract

The big-head schizothorcin (*Aspiorhynchus laticeps*) is an endemic and near-extinction freshwater fish in Xinjiang, China. In this study, a chromosome-scale genome assembly of *A. laticeps* was generated using PacBio and Hi-C techniques. The PacBio sequencing data resulted in a 1.58 Gb assembly with a contig N50 of 1.27 Mb. Using Hi-C scaffolding approach, 88.38% of the initial assembled sequences were anchored and oriented into a chromosomal-scale assembly. The final assembly consisted of 25 pseudo-chromosomes that yielded 1.37 Gb of sequence, with a scaffold N50 of 44.02 Mb. BUSCO analysis showed a completeness score of 93.7%. The genome contained 48,537 predicted protein-coding genes and 58.31% of the assembly was annotated as repetitive sequences. Whole genome duplication events were further confirmed using 4dTv analysis. The genome assembly of *A. laticeps* should be valuable and important to understand the genetic adaptation and endangerment process of this species, which could lead to more effective management and conservation of the big-head schizothorcin and related freshwater fish species.

## Abstract (summary)

**Table:** 

Measurement(s)	Genome Assembly Sequence
Technology Type(s)	Next Generation Sequencing
Sample Characteristic - Organism	*Aspiorhynchus laticeps*
Sample Characteristic - Location	Xinjiang Autonomous Region

## Background & Summary

Freshwater fish can not only provide sufficient food resources for people living in inland region, but also play crucial roles in maintaining ecological balance. However, limited knowledge of freshwater biodiversity has largely constrained effective conservation efforts and public concern^1^, especially for places considered as biodiversity hotspots^2^. Family Cyprinidae (minnows and carps) is a group of common freshwater fish family with abundant and diversified species (more than 1600 species), which dominated flowing waters and lakes worldwide^3^. Cyprinids are known to the public due to some small fish species or farmed carps, yet whether some large and elusive species are under proper management and conservation attentions is uncertain^1^.

**Fig. 1:**
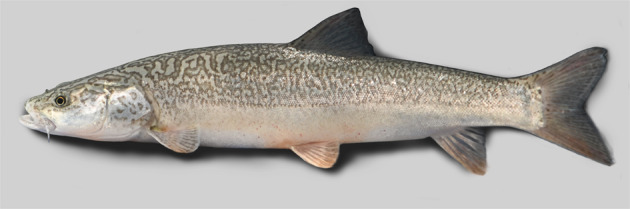
A picture of the big-head schizothorcin used in the genome sequencing and assembly (age: 4 Years, total length: 48.60 cm).

The big-head schizothorcin (*Aspiorhynchus laticeps*) (Fig. 1), also known as Xinjiang datou fish, is a large-size cyprinid (maximum total length 200 cm) endemic to Xinjiang Uygur Autonomous Region of China^1,4^. *A. laticeps* was one of the main targets in local fishery industry with high commercial value^1,5^. As one of the top predators in local environment, this species also has high ecological value for maintaining ecosystem stability^6^. Apart from high commercial and ecological value, with an evolutionary history of nearly 300 million years, *A. laticeps* is also a unique and ideal candidate for phylogeny and evolution studies of schizothorcins^5^. However, due to slow growth rate, long life span and low reproductive rate, as well as the adverse impacts of overexploitation and habitat degradation, the population resources of *A. laticeps* were drastically declined in the 1980s^1,4^. As a result, this species was listed as an endangered species in the China Red Book of endangered animals in 1998^7^. In recent years, based on distribution survey information, regional fish biologists believed that this species might be near extinction^4^. Effective management and conservation strategies are urgently needed for prosperity of *A. laticeps*.

Previous studies generally focused on biology and physio-ecology of *A. laticeps*^5,8–10^, yet limited genetic resources and a lack of genomic information have largely constrained conservation genetics of this species. Therefore, genetic information such as the degree of genetic diversity, evolutionary history and genetic basis of endangerment process, which could provide valuable reference information for resource management and conservation of *A. laticeps*, are still unknown. The development of sequencing techniques and genome-scale analytical approaches have greatly broadened our understanding of genetic adaptation and endangerment process of threatened animals^11^ including panda^12^, Baiji dolphin^13^ and finless porpoise^14^, leading to more effective management and conservation of these species.

**Fig. 2:**
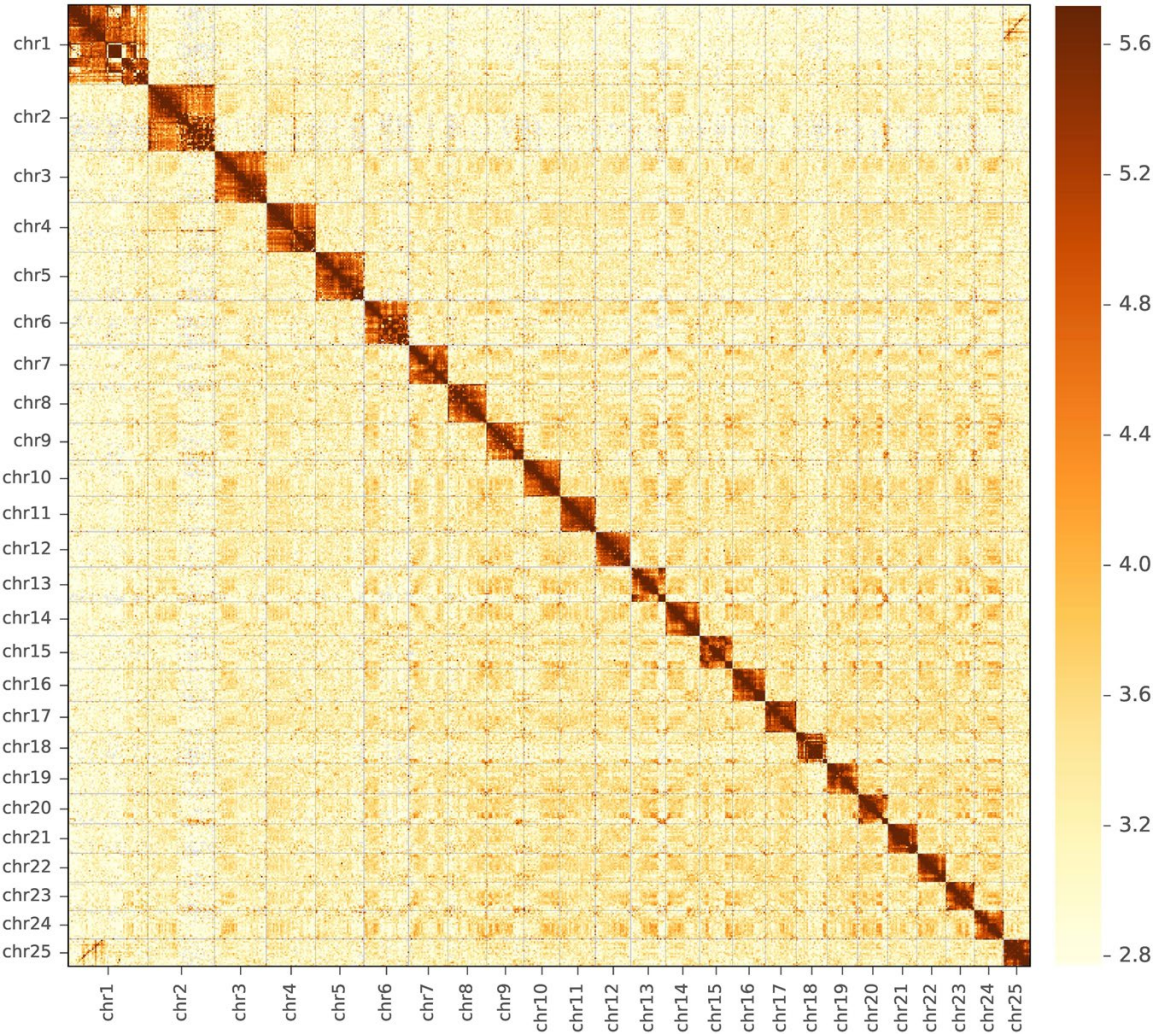
The Hi-C contact map of the *A. laticeps* genome. chr 1–25 represented for the 25 pseudo-chromosomes. The color bar showed the contact density from white (low) to black (high).

**Fig. 3:**
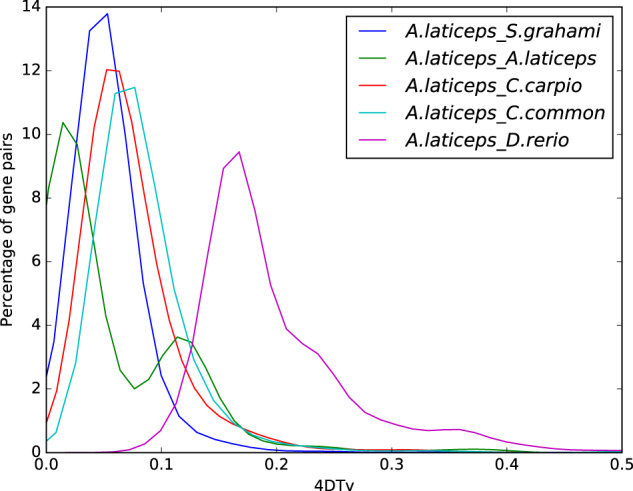
Analysis of fourfold synonymous third-codon transversion (4dTv) indicated whole genome duplication events.

In this study, we assembled a chromosome-scale genome sequence of *A. laticeps* using Illumina short reads, PacBio long reads and Hi-C techniques (Table 1; Fig. 2). The initial genome assembly had a total length of 1,582.9 Mb with 4,133 contigs and a contig N50 of 1.27 Mb (Table 2). After Hi-C scaffolding approach, 88.38% of the initial assembled sequences were anchored to 25 pseudo-chromosomes (according to our results of karyotype analysis), and the total length of the final genome assembly was 1,366.83 Mb, with 3,067 scaffolds and a scaffold N50 of 44.02 Mb (Table 2). In our assembled sequence, a total of 923.02 Mb of repetitive sequences were annotated, representing 58.31% of the genome assembly (Table 2). The repetitive sequences (Table 3) were dominated by DNA transposons (283.13 Mb, 17.89%), long interspersed elements (LINEs, 152.00 Mb, 9.60%) and long terminal repeats (LTRs, 93.17 Mb, 5.89%). In addition, combining *ab initio*, homology-based and Iso-Seq assisted gene prediction approaches, a total of 48,537 protein-coding genes were predicted, among which 47,211 (97.27%) were annotated (Table 2). Such large number of protein-coding genes suggested potential whole genome duplication (WGD) event of *A. laticeps*. Subsequently, analysis of fourfold synonymous third-codon transversion (4dTv) confirmed two major WGD events of *A. laticeps* (Fig. 3). The assembled genome sequences provide useful and valuable information for elucidating the genetic adaptation and underlying molecular basis of endangerment process of *A. laticeps*, which can facilitate to establish more effective management and conservation strategies of this species. These genomic data can be also used in future comparative genomics and phylogenomics studies to investigate genomic evolution and phylogeny of schizothorcins.

**Table 1:** Sequencing data for the *A. laticeps* genome assembly.

Sequencing technology	Illumina	PacBio	Hi-C	Iso-Seq
Library size (bp)	350	20,000	350	3000
Raw data (Gb)	173.68	327.44	155.7	90.67
Clean data (Gb)	169.69	—	143.01	—
Coverage (X)^†^	106.06	204.65	89.38	—
Mean read length (bp)	144	18,225.38	144	1955.65

^†^The coverage was calculated using an estimated genome size of 1.6 Gb.

**Table 2:** Assembly and annotation statistics of the *A. laticeps* genome.

	Total length (Mb)	N_contig	Contig N50	N_scaffold	Scaffold N50
PacBio sequencing	1,584,292,485	4,133	1,266,850	—	—
Hi-C sequencing	1,366,832,638	—	—	3,067	44,016,701
*Genome annotation*					
Protein-coding gene	48,537 (47,211 annotated, 97.27%)				
Repetitive sequence	58.31%				
GC content	37.99%				

Note: N_contig and N_scaffold denote number of contig and number of scaffold respectively.

**Table 3:** Statistics of repetitive sequences in the *A. laticeps* genome.

	Repeat size (bp)	Percentage of genome (%)
*Identification method*		
RepeatMasker	479,782,511	30.31
ProteinMask	227,512,931	14.37
De novo	804,212,276	50.81
TRF	97,644,119	6.17
Total	923,021,458	58.31
*Biological classification*		
DNA	283,131,445	17.89
LINE	151,999,356	9.6
SINE	8,091,895	0.51
LTR	93,174,051	5.89
Unknown	516,278,379	32.62
Other	115,288,798	7.28
Total	903,156,224	57.06

## Methods

### Sample collection and sequencing

A 4 years old *A. laticeps* individual was sampled from Scientific Observing and Experimental Station of Fishery Resources and Environment in Northwest China in April 2020. All experimental methods were performed according to relevant guidelines and regulations established by the Institutional Animal Care and Use Committee of Xinjiang Fisheries Research Institute and Xinjiang Uygur Autonomous Region Aquatic Bureau. The muscle tissue below the dorsal fin was taken and stored in the liquid nitrogen until DNA extraction. Genomic DNA was isolated using the cetyltrimethylammonium bromide (CTAB) method. High-quality DNA was used for library preparation and high-throughput sequencing.

Illumina short-insert (350 bp) libraries were prepared according to the protocol and paired-end (PE150) sequenced on the Illumina Novaseq 6000 platform (Illumina, Inc., San Diego, CA, USA). Long-read sequencing was performed using the PacBio Sequel II sequencer (Pacific Biosciences, Menlo Park, CA, USA). For Hi-C sequencing, fresh muscle was fixed with formaldehyde in a concentration of 1% and the fixation was terminated using 0.2 M glycine. A Hi-C library was prepared following the Hi-C library protocol^15^ and then sequenced using an Illumina Novaseq 6000 sequencing platform. The heart, liver and muscle tissues were pooled for full-length Iso-Seq on the PacBio Sequel II sequencing platform.

### Genome assembly

A total of 173.68 Gb Illumina short-read data were generated. After quality control by using HTQC v1.92.3^16^, clean data were utilized for genome size estimation (Table 1). *K*-mer analysis was conducted using Jellyfish v2.2.10^17^. The *k* value was set to 21 and the genome size was estimated to be 1676.07 Mb, with a heterozygosity ratio of 0.78% and repeat sequence ratio of 76.67%. A total of 327.44 Gb PacBio long-read data (Table 1) were used for *de novo* genome assembly using Wtdbg2^18^ and the draft contigs were corrected using Arrow v2.2.1^19^ with the same PacBio dataset. The Illumina short reads (clean data 169.69 Gb, Table 1) from the same individual were further used to polish the initial genome assembly using Pilon v1.23^20^ (parameters:–frags;–fix snp,indels;–vcf). These sequencing data resulted in a 1,582.9 Mb assembly with 4,133 contigs and a contig N50 of 1.27 Mb (Table 2). The draft genome contigs were then anchored and oriented into a chromosomal-scale assembly using the Hi-C data. A total of 143.01 Gb clean data (Table 1) were aligned to the draft genome assembly using BWA v0.7.10^21^. Duplication removal, sorting, and quality control were performed using HiC-Pro v2.8.0^22^. Only uniquely mapped valid read pairs were used for further analysis. LACHESIS^23^ was then used to cluster, order, and orient the contigs into chromosomal-scale assembly. Finally, 88.38% of the initial assembled sequences were anchored to 25 pseudo-chromosomes (Fig. 2) with lengths ranging from 34.35 to 100.46 Mb, and the total length of the genome assembly was 1,366.83 Mb, with 3,067 scaffolds and scaffold N50 of 44.02 Mb (Table 2).

### Repetitive sequence annotation

A combined strategy based on homology alignment and *de novo* search was applied in our repeat annotation pipeline. A *de novo* repetitive elements database was built by LTR_FINDER^24^, RepeatScout^25^, RepeatModeler (www.repeatmasker.org/RepeatModeler.html) with default parameters. Tandem repeats were also *ab initio* extracted using TRF v4.09^26^. Then all repeat sequences with lengths >100 bp and gap ‘N’ less than 5% constituted the raw transposable element (TE) library. The homolog-based predictions were searched against Repbase^27^ database employing RepeatMasker v3.3.0^28^ software and its in-house scripts RepeatProteinMask (v3.2.2) with default parameters. The combination of Repbase and our *de novo* TE library was processed by uclust^29^ to yield a non-redundant library and RepeatMasker was used to identify DNA-level repeat. The results of repetitive sequence annotation are listed in Table 3.

### Protein-coding gene prediction and annotation

We employed *ab initio*, homology-based and Iso-Seq assisted prediction to detect the protein-coding genes. For homology-based prediction, protein sequences of *Cyprinus carpio, Carassius auratus* common *and C. auratus* red were downloaded from GenBank and Ensembl database^30^. The protein sequences were aligned against the genome assembly using TBLASTN v2.2.26^31^ (E-value ≤ 1e-5), and then matching proteins were aligned to the homologous genome sequences for accurate spliced alignments with GeneWise v2.4.1^32^. The *ab initio* prediction was performed using Augustus v3.2.3^33^, GeneID v1.4^34^, GENESCAN v1.0^35^, GlimmerHMM v3.04^36^, and SNAP v2013-11-29^37^ based on the repeat masked genome sequences. The Iso-Seq data were processed using SMRTlink v5.0 (PacBio, Menlo Park, CA) (parameters: min_length 200; max_drop_fraction 0.8; no_polish TRUE; min_zscore −9999; min_passes 1; min_predicted_accuracy 0.8; max_length 18000) to obtain full-length non-chimeric (FLNC) reads. The FLNC reads were then aligned to the genome using GMAP^38^ with parameters (–no-chimeras;–cross-species;–expand-offsets 1; -B 5; -K 50000; -f samse; -n 1), and then coding regions were predicted using PASA^39^ and GeMoMa v1.7.1^40^. Finally, genes predicted by the above three methods were merged into a non-redundant reference gene set with EvidenceModeler v1.1.1^41^ with identical weights, leading to a total of 48,537 protein-coding genes (Table 2).

Protein-coding genes were annotated by aligning the gene sequences to the SwissProt, NT, NR, Gene Ontology (GO) and Kyoto Encyclopedia of Genes and Genomes (KEGG) databases using BLAST + v2.2.28^42^ with an e-value threshold of 1e-5. InterProScan v5.31^43^ was used to predict protein function based on conserved domains and motif by searching against ProDom, PRINTS, Pfam, SMRT, PANTHER and PROSITE. Ultimately, 47,211 (97.27%) predicted genes were successfully annotated (Table 2).

The predicted gene number was comparable with *C. carpio* and *C. auratus*, which was almost twice that detected in zebrafish *Danio rerio*^44^. Such large number of protein-coding genes suggested potential whole genome duplication (WGD) events of *A. laticeps*. To verify the WGD events, we performed analysis of fourfold synonymous third-codon transversion (4dTv). Syntenic blocks were identified using MCscan v0.8^45^ and homologous protein sequences from these syntenic blocks were aligned using MUSCLE^46^, and the 4dTv values were calculated in PAML package^47^. The 4dTv results also confirmed two major WGD events of *A. laticeps* (Fig. 3).

## Data Records

The sequencing dataset and genome assembly were deposited in public repositories. Illumina, PacBio, Hi-C and RNA-seq sequencing data used for Genome assembly have been deposited in the Genome Sequence Archive (GSA) at the National Genomics Data Center (NGDC)/China National Center for Bioinformation (CNCB) under accession number CRA006604^48^. The whole genome sequence data reported in this paper have been deposited in the National Center for Biotechnology Information (NCBI) GenBank database under the accession JALXFT000000000.1^49^. Moreover, the genomic annotation results have been deposited at the Figshare database^50^.

## Technical Validation

### Evaluation of the quality of genomic DNA and RNA

In our DNA extraction section, the DNA quality and concentration were measured using agarose gel electrophoresis (1%), pulse field gel electrophoresis (1%) and Qubit 3.0 (Thermo Fisher Scientific, Inc., Carlsbad, CA, USA), respectively. For RNA, the integrity and quantity was evaluated using the Agilent 2100 Bioanalyzer (Agilent, USA). Subsequently, high-quality DNA and RNA were used for library preparation and high-throughput sequencing.

### Evaluation of the completeness of genome assembly

The contamination evaluation of assembled genome sequence was performed against the NT database using BLAST+ v2.2.28^41^ with an e-value threshold of 1e-5. The results showed that no bacterial or artificial contaminants in our assembled genome. The completeness of the assembled genome sequence was evaluated using BUSCO v3.0.1^51^. The BUSCO analysis against the vertebrata_odb10 database found that 95.7% of the conserved single copy orthologue genes, including 93.7% of the complete and 2.0% fragmented genes, were found in the genome assembly (Table 4). Also, the mapping rate of Illumina short reads from same individual were further used to evaluate the quality of the initial genome assembly using BWA v0.7.10^21^. By using a total of 169.69 Gb Illumina sequencing data from the same individual, the mapped read rate was 97.88% (Table 4), showing high genome assembly quality.

**Table 4:** Genome quality assessment statistics of the *A. laticeps* genome.

Genomic characteristic	Percentage
Illumina reads mapping rate	97.88%
Illumina reads coverage	98.73%
BUSCO evaluation	n = 3,354
Complete BUSCOs	3,144 (93.7%)
Complete and single-copy BUSCOs	2,289 (68.2%)
Complete and duplicated BUSCOs	855 (25.5%)
Fragmented BUSCOs	68 (2.0%)
Missing BUSCOs	142 (4.3%)

## Data Availability

All software used in this study are in the public domain, with parameters being clearly described in Methods. If no detail parameters were mentioned for the software, default parameters were used as suggested by developer.
